# Reevesioside A, a Cardenolide Glycoside, Induces Anticancer Activity against Human Hormone-Refractory Prostate Cancers through Suppression of c-myc Expression and Induction of G1 Arrest of the Cell Cycle

**DOI:** 10.1371/journal.pone.0087323

**Published:** 2014-01-27

**Authors:** Wohn-Jenn Leu, Hsun-Shuo Chang, She-Hung Chan, Jui-Ling Hsu, Chia-Chun Yu, Lih-Ching Hsu, Ih-Sheng Chen, Jih-Hwa Guh

**Affiliations:** 1 School of Pharmacy, National Taiwan University, Taipei, Taiwan; 2 Graduate Institute of Natural Products, College of Pharmacy, Kaohsiung Medical University, Kaohsiung, Taiwan; National Health Research Institutes, Taiwan

## Abstract

In the past decade, there has been a profound increase in the number of studies revealing that cardenolide glycosides display inhibitory activity on the growth of human cancer cells. The use of potential cardenolide glycosides may be a worthwhile approach in anticancer research. Reevesioside A, a cardenolide glycoside isolated from the root of *Reevesia formosana*, displayed potent anti-proliferative activity against human hormone-refractory prostate cancers. A good correlation (r^2^ = 0.98) between the expression of Na^+^/K^+^-ATPase α_3_ subunit and anti-proliferative activity suggested the critical role of the α_3_ subunit. Reevesioside A induced G1 arrest of the cell cycle and subsequent apoptosis in a thymidine block-mediated synchronization model. The data were supported by the down-regulation of several related cell cycle regulators, including cyclin D1, cyclin E and CDC25A. Reevesioside A also caused a profound decrease of RB phosphorylation, leading to an increased association between RB and E2F1 and the subsequent suppression of E2F1 activity. The protein and mRNA levels of c-myc, which can activate expression of many downstream cell cycle regulators, were dramatically inhibited by reevesioside A. Transient transfection of c-myc inhibited the down-regulation of both cyclin D1 and cyclin E protein expression to reevesioside A action, suggesting that c-myc functioned as an upstream regulator. Flow cytometric analysis of JC-1 staining demonstrated that reevesioside A also induced the significant loss of mitochondrial membrane potential. In summary, the data suggest that reevesioside A inhibits c-myc expression and down-regulates the expression of CDC25A, cyclin D1 and cyclin E, leading to a profound decrease of RB phosphorylation. G1 arrest is, therefore, induced through E2F1 suppression. Consequently, reevesioside A causes mitochondrial damage and an ultimate apoptosis in human hormone-refractory prostate cancer cells.

## Introduction

Cardenolide glycosides, a class of steroid-like compounds, are well appreciated in the treatment of congestive heart failure and arrhythmia. The mechanism of action arises from the inhibition of Na^+^/K^+^-ATPase, leading to an increase of intracellular Ca^2+^ concentrations [1]. Cardenolide glycosides have a narrow therapeutic index that limits the broader application to treat other diseases. However, it has been documented recently that the anticancer activities induced by cardenolide glycosides occurred at concentrations that are achievable in humans without toxic effects. It has been suggested that this class of compounds may be useful for anticancer treatment [1]–[3]. Within the past decade, a variety of studies have identified the anti-proliferative effects of cardenolide glycosides in human malignant tumor cells. The diverse mechanisms have been reported to be involved in the exposure to this class of compounds, including the increase of intracellular Ca^2+^ concentrations [4], oxidative stress [4], [5] and mitochondrial injury [6], the increase of FasL expression [7], [8], inhibition of transcription factors NF-κB and AP-1 [8], suppression of Akt activity [9], down-regulation of protein levels of Bcl-2 and Bcl-xL [10], and inhibition of topoisomerases [11]. Although Na^+^/K^+^-ATPase is the primary target of cardenolide glycosides, not all of the identified mechanisms that lead to the inhibition of cell proliferation are relevant to the suppression of the pumping activity of the enzyme.

Na^+^/K^+^-ATPase consists of two types of subunits, α and β, and a single transmembrane spanning protein FXYD — the conserved amino acids in its signature motif Phe-Xxx-Tyr-Asp [1]. The α subunit is the catalytic subunit of the enzyme and is responsible for the binding of Na^+^, K^+^ and ATP [1], [12]. The β subunit has been suggested to serve as an adhesion molecule and regulate gap junction proteins, the holoenzyme maturation and the transport of α subunit to plasma membrane [1], [12], [13]. The conjugation of cardenolide glycosides with α subunits results in the inhibition of ATP binding and the blockade of enzyme activity in exchanging intracellular Na^+^ and extracellular K^+^. The accumulation of intracellular Na^+^ concentrations, in turn, triggers the influx of calcium into cells [12], [13].

A variety of human cancers have been identified to express different levels of subunit isoforms of Na^+^/K^+^-ATPase, in which certain isoforms have been demonstrated to be up-regulated in specific cancers, including pancreatic cancers, colon cancers, non-small-cell-lung cancers, glioblastomas, melanomas and prostate cancers [14]–[16]. Several studies provide evidence that particular Na^+^/K^+^-ATPase isoforms are crucial in the progression of epithelial-to-mesenchymal transition in cancers [17]. Moreover, a lot of patients fail to respond to cancer chemotherapy due to intrinsic resistance in cancer cells or acquisition of multidrug resistant phenotype during chronic treatment. Several reports suggest that the ligands that target Na^+^/K^+^-ATPase and inhibit the enzyme activity can combat the resistance in cancer cells [15], [18]. Therefore, it has been suggested that Na^+^/K^+^-ATPase can serve as a target for anticancer therapy. Currently, several cardenolide glycoside-based anticancer drugs are under clinical trials [2], [3].

*c-myc* is a regulator gene which codes for a transcription factor. c-myc mutation has been identified in a variety of cancers that results in constitutive expression of this transcription factor, leading to uncontrolled expression of many downstream genes involved in cell proliferation and, ultimately, in cancer formation [19]. Malfunctions in c-myc have been found in numerous cancers, including lymphoma, lung cancers, breast cancers, colon cancers, gastric cancers and prostate cancers [19]–[22]. The c-myc proto-oncogene contributes to various cellular processes including cell proliferation, apoptosis, differentiation and angiogenesis [19]. A large body of evidence supports that c-myc is a promising target for anticancer approach [19]–[22].

Recently, bioassay-guided fractionation of the root of *Reevesia formosana* led to the isolation of new cardenolide glycosides in our work [23]. The determination of the anticancer activity against prostate cancers in the present study showed that reevesioside A displayed potent activity in blocking c-myc expression and inducing arrest of the cell cycle as well as cell apoptosis. The signaling pathways following the exposure to reevesioside A has been identified to demonstrate the anticancer potential of this natural product in prostate cancers.

## Materials and Methods

### Materials

RPMI 1640 medium and fetal bovine serum (FBS) were obtained from GIBCO/BRL Life Technologies (Grand Island, NY). Antibodies to cyclin D1, cyclin E, cyclin A, cyclin B1, cyclin-dependent kinase 4 (Cdk4), Cdk2, PARP, E2F1, CDC25A, α-tubulin, Bcl-2, Bcl-xL, Mcl-1, Bak, Bid, Bax, Bad, Na^+^/K^+^-ATPase α_3_ subunit, c-myc (N262), c-myc siRNA and anti-mouse and anti-rabbit IgGs were obtained from Santa Cruz Biotechnology, Inc. (Santa Cruz, CA). Antibodies to Cdk1, retinoblastoma (RB), p-RB^Ser801/811^, caspase-8, caspase-9, caspase-3, caspase-7, p-Akt^Ser473^, p-Akt^Thr308^, Akt, c-myc, acetyl-α-tubulin and GAPDH were from Cell Signaling Technologies (Boston, MA). Sulforhodamine B (SRB), propidium iodide (PI), phenylmethylsulfonylfluoride (PMSF), trichloroacetic acid (TCA), CGP-37157 and all other chemical compounds were obtained from Sigma-Aldrich (St. Louis, MO). Fluo-3/AM and carboxyfluorescein succinimidyl ester (CFSE) were from Molecular Probes Inc. (Eugene, OR, USA). Reevesioside A was isolated from the root of *reevesia formosana*. The purification and identification of reevesioside A were published elsewhere [23].

### Cell lines and cell culture

Human hormone-refractory prostate cancer (HRPC) cell lines PC-3 and DU-145 were from American Type Culture Collection (Rockville, MD). Cells were cultured in RPMI 1640 medium with 10% FBS (v/v) and penicillin (100 U/ml)/streptomycin (100 µg/ml). Cultures were maintained in a humidified incubator at 37°C in 5% CO_2_/95% air.

### SRB assays

Cells were seeded in 96-well plates in medium with 5% FBS. After 24 hours, cells were fixed with 10% TCA to represent cell population at the time of drug addition (T_0_). After additional incubation of DMSO or the compound for 48 hours, cells were fixed with 10% TCA and SRB at 0.4% (w/v) in 1% acetic acid was added to stain cells. Unbound SRB was washed out by 1% acetic acid and SRB bound cells were solubilized with 10 mM Trizma base. The absorbance was read at a wavelength of 515 nm. Using the following absorbance measurements, such as time zero (T_0_), control growth (C), and cell growth in the presence of the compound (Tx), the percentage growth was calculated at each of the compound concentrations levels. Percentage growth inhibition was calculated as: [1-(Tx-T_0_)/(C-T_0_)] x 100%. Growth inhibition of 50% (IC_50_) is determined at the compound concentration which results in 50% reduction of total protein increase in control cells during the compound incubation.

### Cell proliferation assay with CFSE labeling

CFSE was dissolved in DMSO to constitute a storage solution of 10 mM and kept at -80°C until use. The cells were adjusted to a density of 10^6^ cells/ml and were treated with CFSE at a final concentration of 10 µM. After incubation at 37°C for 10 minutes, labeling was blocked by the addition of RPMI medium with 10% FCS. Tubes were placed in ice for 5 minutes and then washed. After centrifugation, the cells were seeded in RPMI medium with 10% FCS for 12, 24 and 48 hours at 37°C under 5% CO_2_/95% air. After the treatment, the fluorescence intensity was determined by flow cytometric analysis.

### Cell cycle synchronization

Synchronization of the cells was performed by thymidine block. Briefly, Cells were treated with 2 mM thymidine in medium/10% FCS for 24 hours. After washing cells with PBS, the block was released by the incubation of cells in fresh medium/10% FCS (indicated as time zero), and cells were harvested at the indicated times. The cell-cycle progression was detected by flow cytometric analysis.

### Flow cytometric analysis of PI staining

After treatment, cells were harvested by trypsinization, fixed with 70% (*v/v*) alcohol at 4°C for 30 minutes and washed with PBS. The cells were centrifuged and resuspended with 0.5 ml PI solution containing Triton X-100 (0.1%, *v/v*), RNase (100 µg/ml) and PI (80 µg/ml). DNA content was analyzed with the FACScan and CellQuest software (Becton Dickinson, Mountain View, CA).

### Measurement of intracellular Ca^2+^ level

After incubation with fluo-3/AM (5 µM) for 30 minutes, cells were washed twice and incubated in fresh medium. Vehicle (0.1% DMSO) or reevesioside A was added to cells and intracellular Ca^2+^ levels were measured by flow cytometric analysis.

### Immunoprecipitation assay

After treatment with vehicle or the indicated agent, the cells were washed twice with ice-cold PBS, lysed in 700 ml of lysis buffer containing 20 mM Tris, pH 7.5, 1 mM MgCl_2_, 125 mM NaCl, 1% Triton X-100, 1 mM PMSF, 10 µg/ml leupeptin, 10 µg/ml aprotinin, 25 mM β-glycerophosphate, 50 mM NaF, and 100 µM sodium orthovanadate, and centrifuged. The supernatant was immunoprecipitated with the antibody against E2F1 in the presence of A/G-agarose beads overnight. The beads were washed four times with lysis buffer for immunoblotting.

### Western blotting

After the treatment, the cells were harvested with trypsinization, centrifuged and lysed in 0.1 ml of lysis buffer containing 10 mM Tris-HCl (pH 7.4), 150 mM NaCl, 1 mM EGTA, 1% Triton X-100, 1 mM PMSF, 10 μg/ml leupeptin, 10 μg/ml aprotinin, 50 mM NaF and 100 μM sodium orthovanadate. Total protein was quantified, mixed with sample buffer and boiled at 90°C for 5 minutes. Equal amount of protein (30 μg) was separated by electrophoresis in SDS-PAGE, transferred to PVDF membranes and detected with specific antibodies. The immunoreactive proteins after incubation with appropriately labeled secondary antibody were detected with an enhanced chemiluminescence detection kit (Amersham, Buckinghamshire, UK).

### Measurement of mitochondrial membrane potential (Δψ_m_)

JC-1, a mitochondrial dye staining mitochondria in living cells in a membrane potential-dependent fashion, was used to determine Δψ_m_. Cells were treated with or without reevesioside A. Thirty minutes before the termination of incubation, the cells were incubated with JC-1 (final concentration of 2 µM) at 37°C for 30 minutes. The cells were finally harvested and the accumulation of JC-1 was determined using flow cytometric analysis.

### Transient transfection

The plasmid encoding Myr-AKT was a gift from Professor Mien-Chie Hung (The University of Texas M. D. Anderson Cancer Center). For transfection, PC-3 cells were seeded into 60-mm tissue culture dishes with 30% confluence and grown for 24 hours to about 50% confluence. Each dish was washed with serum-free Opti-MEM (Life Technologies), and 2 ml of the same medium was added. Aliquots containing Myr-Akt expression vector or a control plasmid in serum-free Opti-MEM were transfected into cells using Lipofectamine 2000 (Invitrogen) following the manufacturer's instructions. After the incubation for 6 hours at 37°C, cells were washed with medium and incubated in 10% FBS-containing RPMI-1640 medium for 48 hours. Then, the cells were treated with or without reevesioside A. The Western blot analyses were performed.

### RNA extraction and reverse transcription polymerase chain reaction (RT-PCR)

Total RNA was extracted (20 μg). The PCR primers pairs used for genes amplification were as follows: c-*myc* forward primer: 5′-TGG TCG CCC TCC TAT GTT G-3′; c-*myc* reverse primer: 5′–CCG GGT CGC AGA TGA AAC TC-3′; *GAPDH* forward primer: 5′-TCC TTG GAG GCC ATG TGG GCC AT-3′; *GAPDH* reverse primer: 5′-TGA TGA CAT CAA GAA GGT GGT GAA G-3′. After denaturation at 94°C for 2 minutes, PCR was performed in a Robocycler Gradient 96 (Stratagene) for 30 cycles. Each reaction cycle includes denaturation at 94°C for 1 minute, annealing at 55°C for 1 minute, and extension at 72°C for 1 minute, followed by a final extension at 72°C for 10 minutes. PCR products were analyzed on 1.5% agarose gel in TAE buffer (40 mM Tris acetate, 1 mM EDTA), and visualized in the presence of 1 μg/ml ethidium bromide staining using BioDoc-It Imaging System (UVP, Upland, CA, USA).

### Data Analysis

Data are presented as the mean±SEM for the indicated number of separate experiments. Statistical analysis of data for multiple groups is performed with one-way analysis of variance. Student's *t*-test is applied for comparison of two groups. *P*-values less than 0.05 are statistically considered significant.

## Results

### Reevesioside A induces anti-proliferative activity in HRPC cells

**Figure 1:**
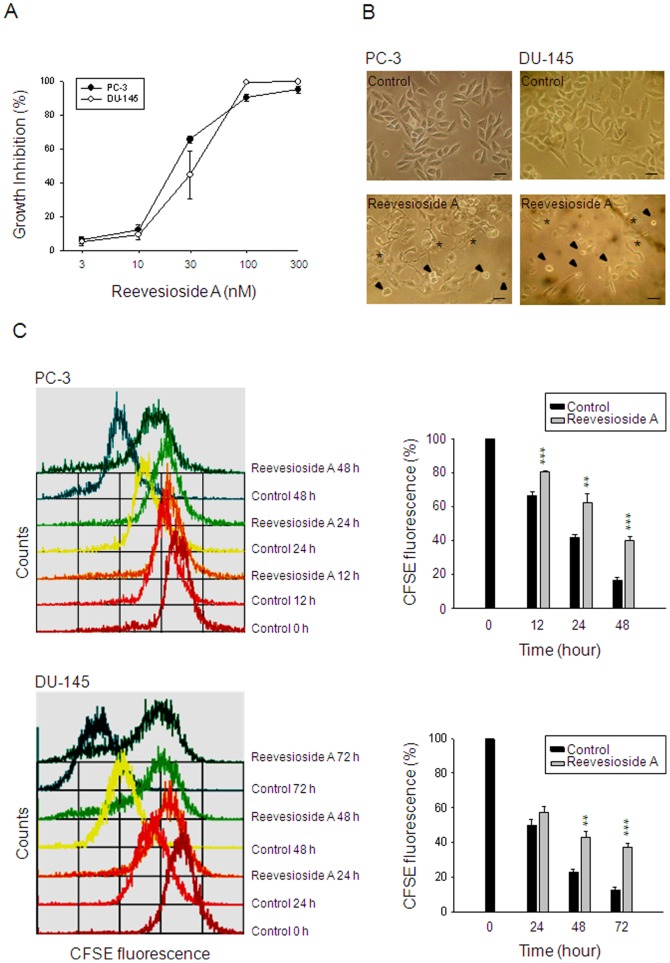
Effect of reevesioside A on cell proliferation. Chemical structure of reevesioside A (A). The graded concentrations of reevesioside A were added to PC-3 and DU-145 cells for 48 hours (A) or a single concentration (50 nM) was added for 48 hours (B) or the indicated times (C). After the treatment, the cells were observed by microscopic examination (B) or the cells were fixed and stained for SRB assay (A) or labeled with CFSE for flow cytometric analysis. Data are expressed as mean±SEM of three to five determinations. ** *P*<0.01 and *** *P*<0.001 compared with the respective control. Arrowhead, cell apoptosis; star, cell differentiation; *bar*, 50 µm.

The SRB assay is used to examine the cell growth based on the measurement of cellular protein content. The data demonstrated that reevesioside A caused a concentration-dependent inhibition of cell growth against HRPC cells, including PC-3 and DU-145, with IC_50_ values of 20.1 and 32.0 nM, respectively (Figure 1A). Microscopic examination showed that reevesioside A induced two different types of cell morphology, the apoptosis with signature of cell shrinkage and the differentiation with spreading morphology in both PC-3 and DU-145 cells (Figure 1B). The cell proliferation was further determined by flow cytometric analysis of CFSE staining. The dye, CFSE, conjugated to cellular proteins and was allocated evenly to daughter cells after cell division. Accordingly, daughter cells showed half the fluorescence intensity of the parents as demonstrated in Figure 1C. Reevesioside A significantly prevented the loss of fluorescence intensity in both PC-3 and DU-145 cells, indicating the inhibition of cell proliferation (Figure 1C).

### Reevesioside A induces G1 arrest of the cell cycle and subsequent apoptosis

**Figure 2:**
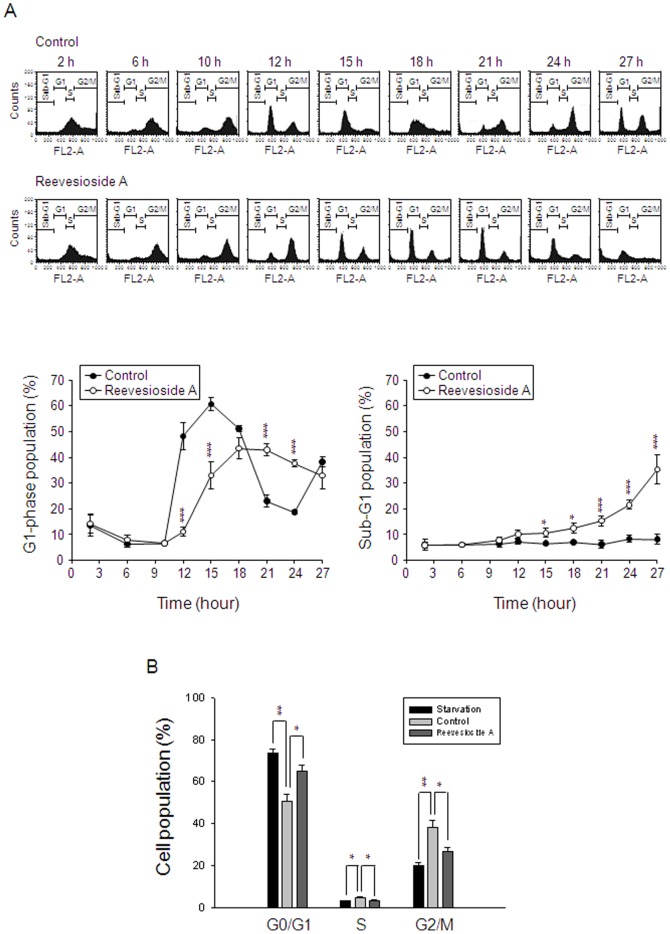
Effect of reevesioside A on cell-cycle progression. (A) Synchronization of PC-3 cells was performed by thymidine block as described in the Materials and Methods section. Then, the cells were released in the absence (upper panel) or presence of 50 nM reevesioside A for the indicated times. Data are representative of five independent experiments. (B) DU-145 cells were incubated in serum-free medium for 48 hours (starvation) and then, 10% FBS was added in the absence or presence of reevesioside A for 18 hours. The cells were harvested for the detection of cell cycle population by flow cytometric analysis. Quantitative data are expressed as mean±SEM of five (A) or three (B) independent experiments. * *P*<0.05, ** *P*<0.01 and *** *P*<0.001 compared with the respective control.

To study the effect of reevesioside A on the progression of cell cycle, PC-3 cells were synchronized predominantly at S phase by using thymidine block treatment. Upon the release from thymidine block in the absence of reevesioside A for six hours, more than 90% of the cells progressed into G2/M phases and then, into G1 phase after the release for 12 to 15 hours. One complete progression of the cell cycle achieved after the release from thymidine block for more than 18 hours (Figure 2A). In the presence of reevesioside A, the progression of cell cycle was delayed and significantly arrested when the cells progressed into G1 phase. Subsequently, apoptotic cell death was triggered in a time-dependent fashion in response to reevesioside A (Figure 2A). Similar effects were detected in DU-145 cells using the method of starvation. The cells were synchronized at G1 phase in serum-free medium for 48 hours. After the release from starvation by 10% FBS supplementation, the cells significantly progressed into S and G2/M phases. Reevesioside A significantly arrested the cells at G1 phase (Figure 2B).

### Reevesioside A induces a profound inhibition of cell cycle regulators

**Figure 3:**
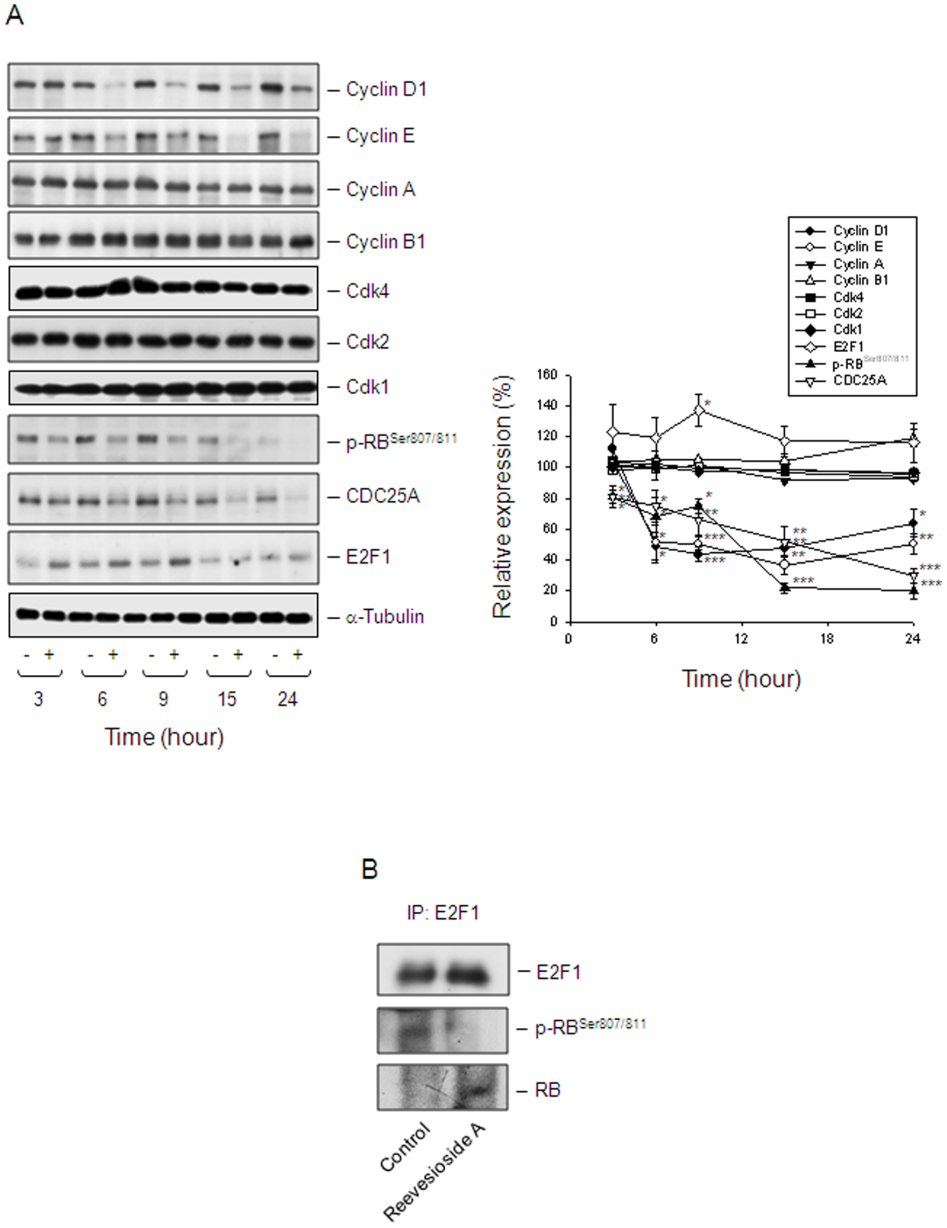
Effect of reevesioside A on the expression of several cell cycle regulators. (A) PC-3 cells were incubated in the absence or presence of reevesioside A (50 nM) for various times. Cells were harvested and lysed for the detection of the indicated protein expression by Western blot analysis. The expression was quantified using the computerized image analysis system ImageQuant (Amersham Biosciences). The data are expressed as mean±SEM of three to five independent experiments. * *P*<0.05, ** *P*<0.01 and *** *P*<0.001 compared with 100% control. (B) After the treatment, the cells were harvested for immunoprecipitation assay. The protein expression was detected by Western blot analysis. Data are representative of three independent experiments.

Cdk activity is regulated by the levels of the cyclin partners and by the association with intrinsic Cdk inhibitors. The cyclin D1/Cdk4 complex is a critical determinant in progression through G1 phase of the cell cycle. The active cyclin D1/Cdk4 complex acts on RB protein for phosphorylation, leading to the release of E2F1 transcription factor which stimulates the expression of G1/S phase genes. In contrast, cyclin E associates with and activates Cdk2. The cyclin E/Cdk2 complex further phosphorylates RB protein, allowing progression of the cell cycle into S phase [24]. After a 6-hour exposure to reevesioside A, the protein levels of both cyclin D1 and cyclin E were dramatically decreased in PC-3 (Figure 3A) and DU-145 cells (Figure S1). The site-specific inhibition of RB phosphorylation at Ser-807/811 by reevesioside A was also tightly associated with the profound decrease of cyclin D1 protein expression in PC-3 (Figure 3A) and DU-145 cells Figure S1). CDC25A, a member of the CDC25 family of dual-specificity phosphatases, is required for the progression from G1 to S phase. The protein levels of CDC25A were down-regulated in a manner similar to that of cyclin D1 (Figure 3A). Altogether, these data were highly correlated with the induction of G1 arrest to reevesioside A action. It was noteworthy that the protein expression of E2F1 was moderately increased by reevesioside A (Figure 3A). The immune-precipitation assay was performed. As a result, reevesioside A prevented the phosphorylation of RB, leading to an increase of the association between RB protein and E2F1 (Figure 3B). The data revealed that E2F1 activity was blocked by the binding of RB.

### Reevesioside A induces mitochondrial dysfunction

**Figure 4:**
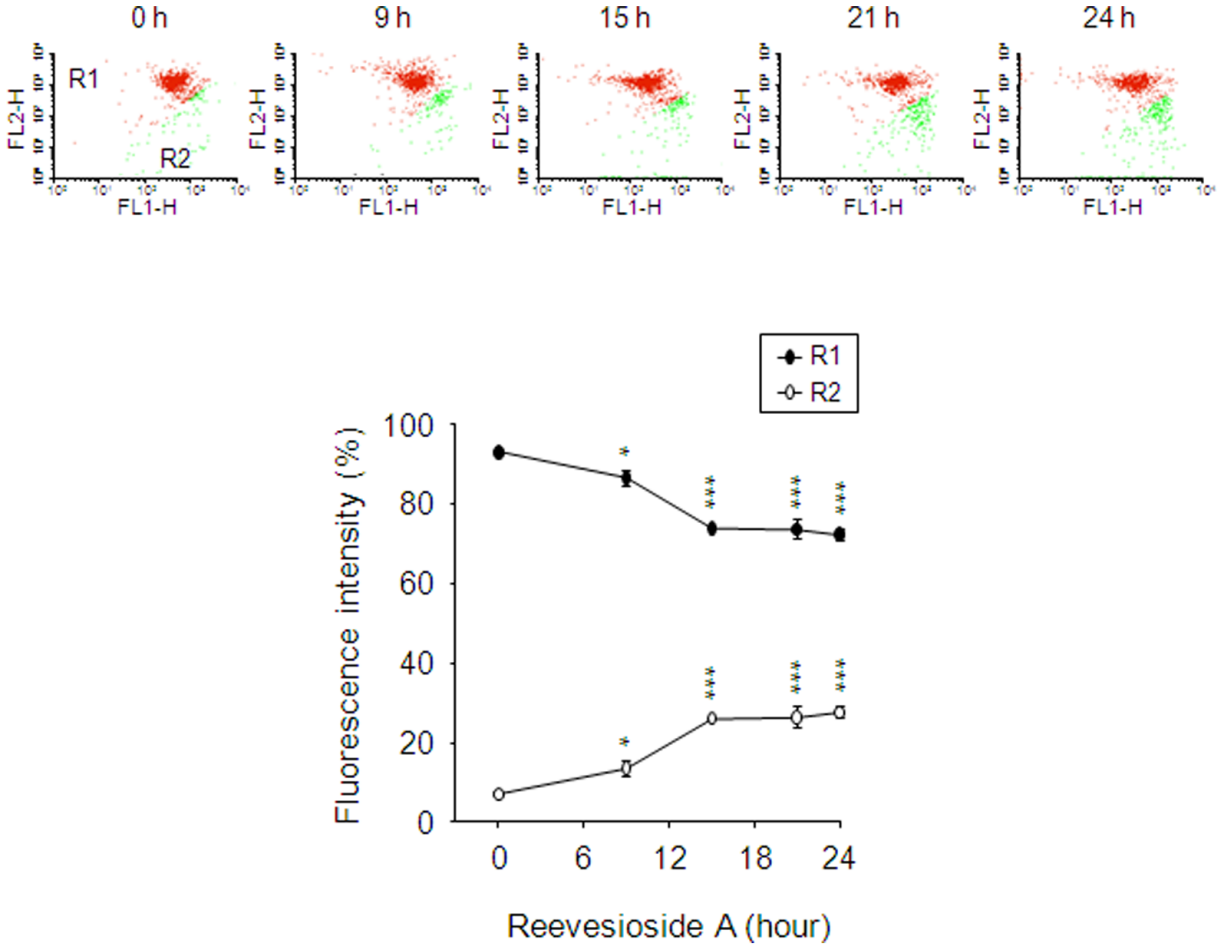
Effect of reevesioside A on mitochondrial membrane potential (Δψ_m_). PC-3 cells were incubated in the absence or presence of reevesioside A (50 nM) for the indicated times. Cells were incubated with JC-1 for the detection of Δψ_m_ using flow cytometric analysis. The data are expressed as mean±SEM of three independent experiments. * *P*<0.05 and *** *P*<0.001 compared with the control.

Mitochondria are involved in various cellular functions, including differentiation, cell signaling, cell growth, cell death and the control of cell cycle [25]. Mitochondria are also well-known serving as a sensor to receive signals from cellular stress that causes arrest of the cell cycle. The Δψ_m_ was examined using JC-1 staining to detect the integrity of mitochondrial membrane. JC-1 aggregates (red fluorescence) favor high Δψ_m_ in intact cells. In response to the loss of Δψ_m_, JC-1 monomers are formed showing green fluorescence. Reevesioside A induced a significant decrease in red fluorescence intensity associated with a concomitant increase in green fluorescence intensity, suggesting the loss of Δψ_m_ and mitochondrial damage in cells (Figure 4).

### Akt is not a functional regulator in reevesioside A-induced effects

**Figure 5:**
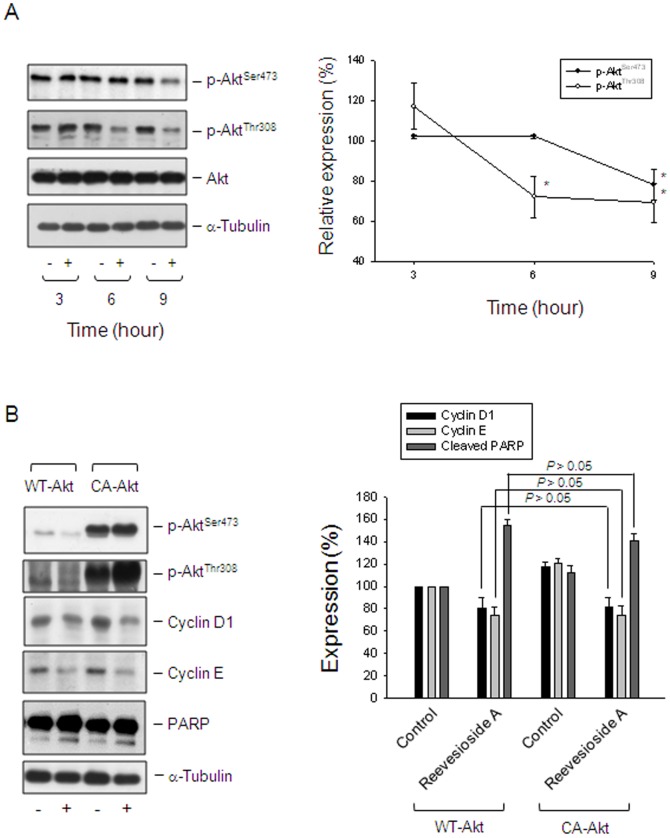
Determination of functional involvement of Akt. (A) PC-3 cells were incubated in the absence or presence of reevesioside A (50 nM) for various times. The cells were harvested and lysed for the detection of the indicated protein by Western blot analysis. (B) PC-3 cells were transfected with the indicated plasmid. Then, the cells were treated without or with reevesioside A (50 nM) for 24 hours. After treatment, the cells were harvested and lysed for the detection of the indicated protein by Western blot analysis. The expression was quantified using the computerized image analysis system ImageQuant (Amersham Biosciences). The data are expressed as mean±SEM of three independent experiments. * *P*<0.05 and ** *P*<0.01 compared with 100% control. WT-Akt, wild type Akt; CA-Akt, constitutively active Akt.

Akt, a serine/threonine kinase, plays a crucial role in regulating cell survival and apoptosis. Akt is activated through the phosphorylation of Thr308 in the activation loop and Ser473 at the carboxyl terminus [26]. Reevesioside A resulted in a decreased phosphorylation in Akt at both Thr308 and Ser473, indicating the inhibition of Akt activity (Figure 5A). To determine the functional role of Akt, PC-3 cells were overexpressed with constitutively active Akt (Myr-Akt) and several expressions including cyclin D1, cyclin E and PARP were detected. Consequently, the overexpression of Myr-Akt neither prevented the down-regulation of both cyclin D1 and cyclin E, nor inhibited the cleavage of PARP (Figure 5B).

### c-myc is an upstream player in reevesioside A-mediated effects

**Figure 6:**
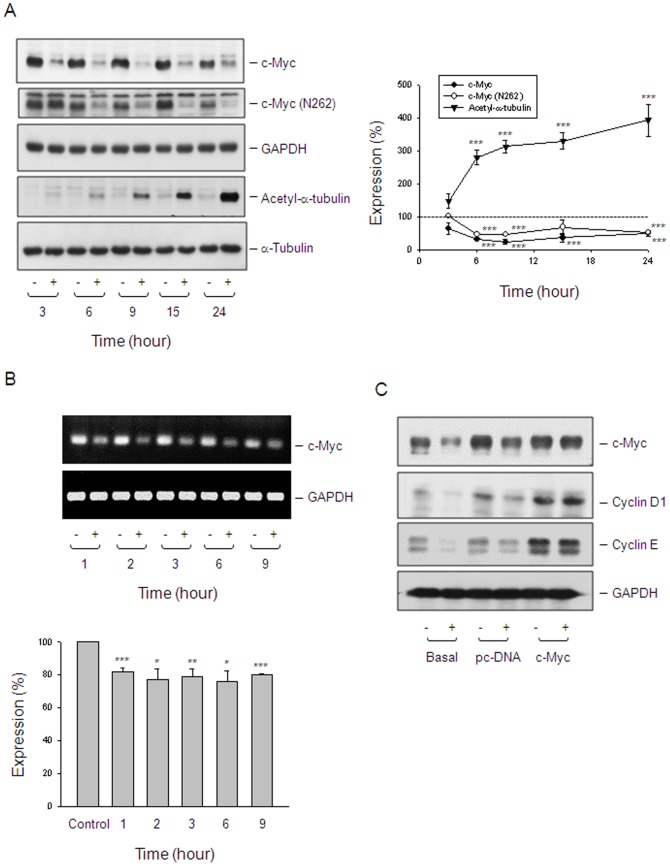
Determination of functional involvement of c-myc. (A) PC-3 cells were incubated in the absence or presence of reevesioside A (50 nM) for various times. The cells were harvested and lysed for the detection of the indicated protein by Western blot analysis. The expression was quantified using the computerized image analysis system ImageQuant (Amersham Biosciences). The data are expressed as mean±SEM of three independent experiments. *** *P*<0.001 compared with 100% control. (B) PC-3 cells were incubated in the absence or presence of reevesioside A (50 nM) for the indicated times. The cells were harvested for the determination of mRNA expression by RT-PCR. (C) PC-3 cells were transfected with the indicated plasmid. The cells were treated without or with reevesioside A (50 nM) for 6 hours. After treatment, the cells were harvested and lysed for the detection of the indicated protein by Western blot analysis.

There are studies showing that activation of c-myc expression is able to trigger quiescent cells entering into cell cycle, whereas blockade of c-myc expression leads to the arrest of the cell cycle [19], [27], suggesting that c-myc plays a critical role in regulating cell cycle. Reevesioside A induced a rapid down-regulation of c-myc protein levels in PC-3 (Figure 6A) and DU-145 cells (Figure S1). The detection of c-myc mRNA levels also demonstrated an inhibitory activity to reevesioside A action (Figure 6B). Further determination of c-myc function by transient transfection of PC-3 cells with c-myc gene resulted in the prevention of protein down-regulation of both cyclin D1 and cyclin E (Figure 6C). The data suggested that c-myc served as an upstream target on reevesioside A-mediated blockade of cell cycle progression. It is noteworthy that reevesioside A caused a time-dependent increase of α-tubulin acetylation in PC-3 (Figure 6A) and DU-145 cells (Figure S1). However, reevesioside A did not inhibit the activity of HDAC using both *in vitro* enzyme assay and cell-based activity assay (data not shown).

### Expression of Na^+^/K^+^-ATPase α_3_ subunit is highly correlated with the anti-proliferative activity

**Figure 7:**
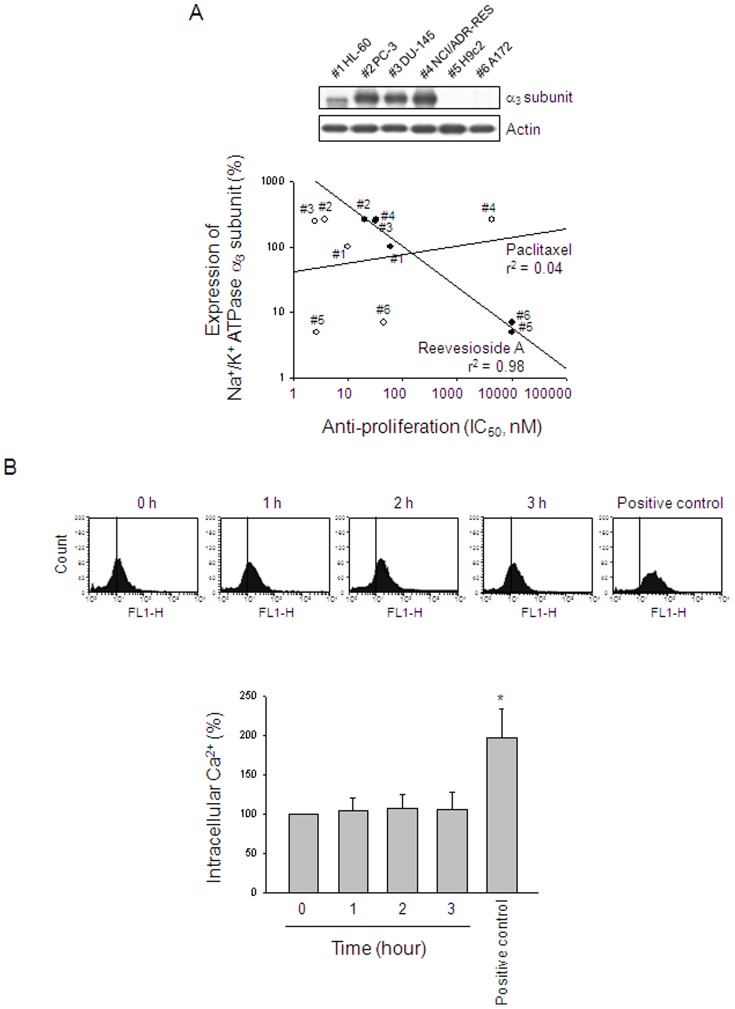
Correlation between Na^+^/K^+^-ATPase α_3_ subunit and anti-proliferative activity. (A) The expression of Na^+^/K^+^-ATPase α_3_ subunit was detected using Western blot analysis. The anti-proliferative IC_50_ values were determined using SRB assays except for HL-60 cells by MTT assays. (B) PC-3 cells were incubated in the absence or presence of reevesioside A (50 nM) for the indicated times. After the treatment, the cells were harvested for the detection of intracellular Ca^2+^ levels using flow cytometric analysis of the staining with fluo-3 AM. Data are expressed as mean±SEM of three independent experiments. * *P*<0.05 compared with the control.

The α subunit is the catalytic subunit of Na^+^/K^+^-ATPase and is responsible for the binding of Na^+^, K^+^ and ATP [1], [12]. To determine whether α_3_ subunit is responsible for reevesioside A-mediated activity, the correlation between the protein expression of α_3_ subunit and anti-proliferative activity of reevesioside A has been conducted in several cancer cell lines, including acute promyelocytic leukemia HL-60, prostate cancer PC-3 and DU-145, multidrug resistant cell line NCI/ADR-RES, cardiomyocyte H9c2 and glioblastoma cell line A172. The data demonstrated a good correlation for reevesioside A but not for paclitaxel, a negative control that causes mitotic arrest of the cell cycle and anti-proliferation through the induction of microtubule stabilization (Figure 7A).

Normally, by inhibiting the Na^+^/K^+^-ATPase, cardenolide glycosides lead to the increase of intracellular sodium concentration which, *in turn*, causes an accumulation of intracellular calcium through Na^+^-Ca^2+^ exchange system [1]. The detection of intracellular calcium by flow cytometric analysis of fluo-3/AM staining showed that reevesioside A did not modify intracellular calcium concentration in PC-3 cells (Figure 7B), indicating that the anticancer effect was not attributed to the Na^+^-Ca^2+^ exchanging activity.

## Discussion

Within the past decade, there has been considerable increase in the number of studies suggesting the anticancer effects of cardenolide glycosides against a wide variety of cancer cells [1]–[10]. It reveals that the use of certain cardenolide glycosides may be a potential approach for the control of cancer cell proliferation even despite their common limitation of narrow therapeutic index. Recently, several promising clinical trials of cardenolide glycosides have been initiated [1]–[3], [28]. Na^+^/K^+^-ATPase, which serves as an energy-transducing ion pump, has been extensively studied for more than fifty years. This enzyme consists of two necessary types of subunits, α and β subunits. The levels of expression of Na^+^/K^+^-ATPase subunits have been reported to be differentially altered in cancer versus normal cells. Both α and β subunit expression levels have been suggested to be predictors of recurrence-free time of bladder cancer patients [29]. The α_1_ subunit has been reported to be actively involved in cancer cell growth and survival [30]. Furthermore, the reduced β subunit levels are associated with the clear-cell renal cancer carcinoma phenotype [31]. Therefore, the altered expression levels of the subunits can be therapeutically targeted to inhibit cancer cell survival. The data in the present study has suggested that α_3_ subunit is critical for reevesioside A-induced anticancer activity. The result is consistent with the studies that α_3_ subunit is responsible for the binding of several cardenolide glycosides [1], [32]. Reevesioside A did not induce any change of intracellular Ca^2+^ concentration using flow cytometric analysis of fluo-3 staining, suggesting that reevesioside A induced anticancer activity through the signaling pathway independent of intracellular Ca^2+^ mobilization.

G1 phase, a cell cycle phase during which the cell grows in size and synthesizes mRNA and proteins for DNA synthesis, is especially important because it determines if a cell commits to division or to escape from the cell cycle. A wide range of cellular stress, such as stress at mitochondria [33], Golgi apparatus [34], endoplasmic reticulum [35] and nucleus [36], has been suggested to interrupt cell cycle progression, leading to G1 arrest. Reevesioside A induced G1 arrest of the cell cycle followed by apoptotic cell death. The G1 arrest was confirmed by a dramatic decrease of protein levels of cyclin D1, a regulatory partner of Cdk4 or Cdk6. The cyclin D1/Cdk4 or cyclin D1/Cdk6 complex activity tightly controls the G1/S transition of the cell cycle through stimulating the phosphorylation of RB protein that, *in turn*, leads to the release of E2F1 transcription factor [37], [38]. E2F1 may display oncogenic activity via stimulating the expression of G1/S phase genes [38]. The immune-precipitation assay showed that under the condition of hypo-phosphorylation of RB to reevesioside A action, the association between RB and E2F1 was increased, leading to the suppression of E2F1 activity and subsequent G1 arrest of the cell cycle. However, reevesioside A also induced an increase of E2F1 protein levels. Several lines of evidence show that E2F1 is stress-responsive. There is study demonstrating that DNA damage may result in the induction of E2F1 accumulation [39]. Furthermore, the anticancer agent flavopiridol induces an increase of E2F1 protein levels that is responsible to the apoptosis in H1299 lung carcinoma cells [40]. These studies indicate that E2F1 may function as a pro-apoptotic factor. Whether E2F1 displays pro-apoptotic activity in reevesioside A-mediated anticancer mechanism warrants further investigation.

Another key regulator for the G1/S transition is CDC25A. CDC25A is able to activate Cdk4 or Cdk6 through removing inhibitory phosphorylation from tyrosine residues. Recent study shows that microRNA-induced silencing of CDC25A abolishes Cdk4/6 capability on association with cyclin D1, blocking downstream cyclin E stimulation and arresting cells at early G1 phase. The study proposes a new approach by using microRNA for anticancer treatment [41]. Similar to the silencing of CDC25A, reevesioside A induced a profound down-regulation of CDC25A, cyclin D1 and cyclin E, facilitating G1 arrest of the cell cycle in prostate cancers.

Akt, a serine/threonine protein kinase, plays a critical role in cell survival and apoptosis. Akt is activated through phospholipid binding and phosphorylation at Thr308 of activation loop. The phosphorylation with the carboxy terminus at Ser473 may also induce Akt activity [26]. Recent studies demonstrate that Na^+^/K^+^-ATPase is also a crucial receptor which transduce ligand binding into the activation of protein kinases. The binding of ouabain, a cardenolide glycoside, to Na^+^/K^+^-ATPase has been reported to induce several intracellular signaling molecules, including ERK1/2 and Akt, which enhance protein translation [42], [43]. The intrinsic Akt activity may impede ouabain-mediated anticancer activity. In contrast, some other cardiac glycosides, such as bufalin and oleandrin, caused the inhibition of Akt activity [44], [45]. Reevesioside A, similar to bufalin and oleandrin, inhibited the Akt activity in PC-3 cells. It is not clear why there exists the discrepancy between these cardiac glycosides on the regulation of Akt activity. Lipid solubility may be one of the reasons since bufalin, oleandrin and reevesioside A are lipid soluble, whereas ouabain is much more water soluble. However, the overexpression of constitutively active Akt neither prevented the down-regulation of both cyclin D1 and cyclin E, nor inhibited the cleavage of PARP, suggesting that the suppression of Akt activity might play a role beyond the regulation of cell cycle and cell apoptosis.

The c*-myc* gene encodes a sequence-specific transcription factor that leads to the expression of numerous genes, some of which are importantly involved in cell proliferation and oncogenesis [19], [22], [46]. On the contrary, c-myc may regulate apoptosis and senescence [47]. The transient transfection of PC-3 cells with c-myc gene was performed to determine if c-myc served as an oncogenic factor or a tumor suppressor. The data revealed that c-myc expression prevented the cells from the down-regulation of both cyclin D1 and cyclin E, suggesting that c-myc played a role on proliferation and oncogenesis. The c-myc also has been suggested to control cell differentiation [48]. Differentiated cancer cells tend to grow at a much slower rate than undifferentiated or poorly differentiated cancer cells that grow uncontrollably. The microscopic examination demonstrated that, under the exposure to reevesioside A, part of the cells showed the differentiated spreading morphology. Demeterco and the colleagues using a β-cell line model have reported that the differentiation is association with a decrease in cell proliferation. The mechanism has been identified that the down-regulation of c-*myc* protooncogene is a critical event. It has been, therefore, suggested that c-myc plays a central role in the switch mechanism by which cell proliferation *vs*. differentiation is determined [48]. The similar mechanism may also explain reevesiosdie A-induced anti-proliferative effect and cell differentiation.

Microtubules are highly dynamic polymers involved in a wide variety of cellular processes, such as cell division, differentiation and signal transduction. Tubulin acetylation is a post-translational modification, which function just begins to be revealed. Tubulin acetylation may positively recruit molecular chaperons and regulate related client proteins involved in cell proliferation and apoptosis [49]. In neural and non-neural cells, tubulin may also form a complex with Na^+^/K^+^-ATPase to regulate the enzyme activity. Increasing lines of evidence suggest that acetylated tubulin, but not non-acetylated form, is able to associate with Na^+^/K^+^-ATPase and to block its catalytic activity [50]. Not only on ATPase activity, tubulin acetylation is also associated with cell apoptosis. Tubacin (tubulin acetylation inducer), a small molecule that selectively inhibits histone deacetylase 6 and causes tubulin acetylation, inhibits cell proliferation and induces apoptosis in numerous types of cancer cells [51]. Reevesioside A induced the acetylation of tubulin in a pattern correlated well with the anti-proliferative signaling as well as the mitochondrial damage stress in PC-3 cell. The tubulin acetylation may play a role on the anticancer activity to reevesioside A action although the mechanism needs further elucidation.

In conclusion, the data suggest that reevesioside A induces anti-proliferative and apoptotic signaling in a sequential manner. The exposure to reevesioside A induces a dramatic down-regulation of c-myc in both mRNA and protein levels which, *in turn*, down-regulates the protein expressions of CDC25A, cyclin D1 and cyclin E. Consequently, RB phosphorylation is inhibited and the association between RB and E2F1 is increased, leading to the suppression of E2F1 activity and subsequent G1 arrest of the cell cycle. Next, reevesioside A induces the loss of Δψ_m_ and mitochondrial damage stress, lead to an ultimate apoptotic cell death.

## Supporting Information

Figure S1 **Effect of reevesioside A on the expression of several proteins in DU-145 cells.** The cells were incubated in the absence or presence of reevesioside A (50 nM) for various times. Cells were harvested and lysed for the detection of the indicated protein expression by Western blot analysis. The expression was quantified using the computerized image analysis system ImageQuant (Amersham Biosciences). The data are expressed as mean±SEM of three independent experiments. * *P*<0.05, ** *P*<0.01 and *** *P*<0.001 compared with 100% control.
(TIF)

## References

[1] Newman RA, Yang P, Pawlus AD, Block KI (2008) Cardiac glycosides as novel cancer therapeutic agents. Mol Interv 8: 36–49. 18332483 10.1124/mi.8.1.8

[2] Haux J, Klepp O, Spigset O, Tretli S (2001) Digitoxin medication and cancer; case control and internal dose-response studies. BMC Cancer 1: 11. 11532201 10.1186/1471-2407-1-11 PMC48150

[3] Vaklavas C, Chatzizisis YS, Tsimberidou AM (2011) Common cardiovascular medications in cancer therapeutics. Pharmacol Ther 130: 177–190. 21277894 10.1016/j.pharmthera.2011.01.009

[4] McConkey DJ, Lin Y, Nutt LK, Ozel HZ, Newman RA (2000) Cardiac glycosides stimulate Ca^2+^ increases and apoptosis in androgen-independent, metastatic human prostate adenocarcinoma cells. Cancer Res 60: 3807–3812. 10919654

[5] Newman RA, Yang P, Hittelman WN, Lu T, Ho DH, et al (2006) Oleandrin-mediated oxidative stress in human melanoma cells. J Exp Ther Oncol 5: 167–181. 16528968

[6] López-Lázaro M (2007) Digitoxin as an anticancer agent with selectivity for cancer cells: possible mechanisms involved. Experts Opin Ther Targets 11: 1043–1053. 10.1517/14728222.11.8.1043 17665977

[7] Raghavendra PB, Sreenivasan Y, Ramesh GT, Manna SK (2007) Cardiac glycoside induces cell death via FasL by activating calcineurin and NF-AT, but apoptosis initially proceeds through activation of caspases. Apoptosis 12: 307–318. 17203245 10.1007/s10495-006-0626-3 PMC2740376

[8] Manna SK, Sah NK, Newman RA, Cisneros A, Aggarwal BB (2000) Oleandrin suppresses activation of nuclear transcription factor-kappaB, activator protein-1, and c-Jun NH2-terminal kinase. Cancer Res 60: 3838–3847. 10919658

[9] Raghavendra PB, Sreenivasan Y, Manna SK (2007) Oleandrin induces apoptosis in human, but not in murine cells: dephosphorylation of Akt, expression of FasL, and alteration of membrane fluidity. Mol Immunol 44: 2292–2302. 17173971 10.1016/j.molimm.2006.11.009

[10] Nasu K, Nishida M, Ueda T, Takai N, Bing S, et al (2005) Bufalin induces apoptosis and the G0/G1 cell cycle arrest of endometriotic stromal cells: a promising agent for the treatment of endometriosis. Mol Hum Reprod 11: 817–823. 16390854 10.1093/molehr/gah249

[11] Watabe M, Nakajo S, Yoshida T, Kuroiwa Y, Nakaya K (1997) Treatment of U937 cells with bufalin induces the translocation of casein kinase 2 and modulates the activity of topoisomerase II prior to the induction of apoptosis. Cell Growth Differ 8: 871–879. 9269896

[12] Schoner W, Scheiner-Bobis G (2007) Endogenous and exogenous cardiac glycosides: their roles in hypertension, salt metabolism, and cell growth. Am J Physiol Cell Physiol 293: C509–536. 17494630 10.1152/ajpcell.00098.2007

[13] Nesher M, Shpolansky U, Rosen H, Lichtstein D (2007) The digitalis-like steroid hormones: new mechanisms of action and biological significance. Life Sci 80: 2093–2107. 17499813 10.1016/j.lfs.2007.03.013

[14] Yang P, Menter DG, Cartwright C, Chan D, Dixon S, et al (2009) Oleandrin-mediated inhibition of human tumor cell proliferation: importance of Na,K-ATPase alpha subunits as drug targets. Mol Cancer Ther 8: 2319–2328. 19671733 10.1158/1535-7163.MCT-08-1085

[15] Mijatovic T, Dufrasne F, Kiss R (2012) Cardiotonic steroids-mediated targeting of the Na^+^/K^+^-ATPase to combat chemoresistant cancers. Curr Med Chem 19: 627–646. 22204337 10.2174/092986712798992075

[16] Li Z, Zhang Z, Xie JX, Li X, Tian J, et al (2011) Na/K-ATPase mimetic pNaKtide peptide inhibits the growth of human cancer cells. J Biol Chem 286: 32394–3403. 21784855 10.1074/jbc.M110.207597 PMC3173162

[17] Rajasekaran SA, Huynh TP, Wolle DG, Espineda CE, Inge LJ, et al (2010) Na,K-ATPase subunits as markers for epithelial-mesenchymal transition in cancer and fibrosis. Mol Cancer Ther 9: 1515–1524. 20501797 10.1158/1535-7163.MCT-09-0832 PMC2884047

[18] Mijatovic T, Kiss R (2013) Cardiotonic steroids-mediated Na^+^/K^+^-ATPase targeting could circumvent various chemoresistance pathways. Planta Med 79: 189–198. 23412992 10.1055/s-0032-1328243

[19] Dang CV (2012) MYC on the path to cancer. Cell 149: 22–35. 22464321 10.1016/j.cell.2012.03.003 PMC3345192

[20] Li Z, Van Calcar S, Qu C, Cavenee WK, Zhang MQ, et al (2003) A global transcriptional regulatory role for c-Myc in Burkitt's lymphoma cells. Proc Natl Acad Sci USA 100: 8164–8169. 12808131 10.1073/pnas.1332764100 PMC166200

[21] Wang J, Kobayashi T, Floc'h N, Kinkade CW, Aytes A, et al (2012) B-Raf activation cooperates with PTEN loss to drive c-Myc expression in advanced prostate cancer. Cancer Res 72: 4765–4776. 22836754 10.1158/0008-5472.CAN-12-0820 PMC3445712

[22] Calcagno DQ, Leal MF, Assumpcao PP, Smith MA, Burbano RR (2008) MYC and gastric adenocarcinoma carcinogenesis. World J Gastroenterol 14: 5962–5968. 18932273 10.3748/wjg.14.5962 PMC2760197

[23] Chang HS, Chiang MY, Hsu HY, Yang CW, Lin CH, et al (2013) Cytotoxic cardenolide glycosides from the root of *Reevesia formosana* . Phytochemistry 87: 86–95. 23313131 10.1016/j.phytochem.2012.11.024

[24] Malumbres M, Barbacid M (2009) Cell cycle, CDKs and cancer: a changing paradigm. Nat Rev Cancer 9: 153–166. 19238148 10.1038/nrc2602

[25] Lindsay J, Esposti MD, Gilmore AP (2011) Bcl-2 proteins and mitochondria–specificity in membrane targeting for death. Biochim Biophys Acta 1813: 532–539. 21056595 10.1016/j.bbamcr.2010.10.017

[26] Wick MJ, Dong LQ, Riojas RA, Ramos FJ, Liu F (2000) Mechanism of phosphorylation of protein kinase B/Akt by a constitutively active 3-phosphoinositide-dependent protein kinase-1. J Biol Chem 275: 40400–40406. 11006271 10.1074/jbc.M003937200

[27] Santoni-Rugiu E, Falck J, Mailand N, Bartek J, Lukas J (2000) Involvement of Myc activity in a G(1)/S-promoting mechanism parallel to the pRb/E2F pathway. Mol Cell Biol 20: 3497–3509. 10779339 10.1128/mcb.20.10.3497-3509.2000 PMC85642

[28] Slingerland M, Cerella C, Guchelaar HJ, Diederich M, Gelderblom H (2013) Cardiac glycosides in cancer therapy: from preclinical investigations towards clinical trials. Invest New Drugs 31: 1087–1094. 23748872 10.1007/s10637-013-9984-1

[29] Espineda C, Seligson DB, James Ball W Jr, Rao J, Palotie A, et al (2003) Analysis of the Na,K-ATPase alpha- and beta-subunit expression profiles of bladder cancer using tissue microarrays. Cancer 97: 1859–1868. 12673711 10.1002/cncr.11267

[30] Lin Y, Ho DH, Newman RA (2010) Human tumor cell sensitivity to oleandrin is dependent on relative expression of Na^+^, K^+^-ATPase subunitst. J Exp Ther Oncol 8: 271–286. 21222360

[31] Rajasekaran SA, Ball WJ Jr, Bander NH, Liu H, Pardee JD, et al (1000) Reduced expression of beta-subunit of Na,K-ATPase in human clear-cell renal cell carcinoma. J Urol 162: 574–580. 10411090

[32] Yang P, Menter DG, Cartwright C, Chan D, Dixon S, et al (2009) Oleandrin-mediated inhibition of human tumor cell proliferation: importance of Na,K-ATPase alpha subunits as drug targets. Mol Cancer Ther 8: 2319–2328. 19671733 10.1158/1535-7163.MCT-08-1085

[33] Chiu CC, Haung JW, Chang FR, Huang KJ, Huang HM, et al (2013) Golden berry-derived 4β-hydroxywithanolide E for selectively killing oral cancer cells by generating ROS, DNA damage, and apoptotic pathways. PLoS One 8: e64739. 23705007 10.1371/journal.pone.0064739 PMC3660349

[34] Lu PH, Chueh SC, Kung FL, Pan SL, Shen YC, et al (2007) Ilimaquinone, a marine sponge metabolite, displays anticancer activity via GADD153-mediated pathway. Eur J Pharmacol 556: 45–54. 17140562 10.1016/j.ejphar.2006.10.061

[35] Han C, Jin L, Mei Y, Wu M (2013) Endoplasmic reticulum stress inhibits cell cycle progression via induction of p27 in melanoma cells. Cell Signal 25: 144–149. 23010535 10.1016/j.cellsig.2012.09.023

[36] Hsu JL, Pan SL, Ho YF, Hwang TL, Kung FL, et al (2011) Costunolide induces apoptosis through nuclear calcium2+ overload and DNA damage response in human prostate cancer. J Urol 185: 1967–1974. 21421237 10.1016/j.juro.2010.12.091

[37] Lundberg AS, Weinberg RA (1998) Functional inactivation of the retinoblastoma protein requires sequential modification by at least two distinct cyclin-cdk complexes. Mol Cell Biol 18: 753–761. 9447971 10.1128/mcb.18.2.753 PMC108786

[38] Harbour JW, Dean DC (2000) The Rb/E2F pathway: expanding roles and emerging paradigms. Genes Dev 14: 2393–2409. 11018009 10.1101/gad.813200

[39] Lin WC, Lin FT, Nevins JR (2001) Selective induction of E2F1 in response to DNA damage, mediated by ATM-dependent phosphorylation. Genes Dev 15: 1833–1844. 11459832 PMC312742

[40] Ma Y, Cress WD, Haura EB (2003) Flavopiridol-induced apoptosis is mediated through up-regulation of E2F1 and repression of Mcl-1. Mol Cancer Ther 2: 73–81. 12533675

[41] Bertero T, Gastaldi C, Bourget-Ponzio I, Mari B, Meneguzzi G, et al (2013) CDC25A targeting by miR-483-3p decreases CCND-CDK4/6 assembly and contributes to cell cycle arrest. Cell Death Differ 20: 800–811. 23429262 10.1038/cdd.2013.5 PMC3647239

[42] Kim SH, Yu HS, Park HG, Ha K, Kim YS, et al (2013) Intracerebroventricular administration of ouabain, a Na/K-ATPase inhibitor, activates mTOR signal pathways and protein translation in the rat frontal cortex. Prog Neuropsychopharmacol Biol Psychiatry 45: 73–82. 23643758 10.1016/j.pnpbp.2013.04.018

[43] Tian J, Li X, Liang M, Liu L, Xie JX, et al (2009) Changes in sodium pump expression dictate the effects of ouabain on cell growth. J Biol Chem 284: 14921–14929. 19329430 10.1074/jbc.M808355200 PMC2685674

[44] Qiu DZ, Zhang ZJ, Wu WZ, Yang YK (2013) Bufalin, a component in Chansu, inhibits proliferation and invasion of hepatocellular carcinoma cells. BMC Complement Altern Med 13: 185. 23870199 10.1186/1472-6882-13-185 PMC3723921

[45] Newman RA, Kondo Y, Yokoyama T, Dixon S, Cartwright C, et al (2007) Autophagic cell death of human pancreatic tumor cells mediated by oleandrin, a lipid-soluble cardiac glycoside. Integr Cancer Ther 6: 354–364. 18048883 10.1177/1534735407309623

[46] Högstrand K, Hejll E, Sander B, Rozell B, Larsson LG, et al (2012) Inhibition of the intrinsic but not the extrinsic apoptosis pathway accelerates and drives MYC-driven tumorigenesis towards acute myeloid leukemia. PLoS One 7: e31366. 22393362 10.1371/journal.pone.0031366 PMC3290626

[47] Uribesalgo I, Benitah SA, Di Croce L (2012) From oncogene to tumor suppressor: the dual role of Myc in leukemia. Cell Cycle 11: 1757–1764. 22510570 10.4161/cc.19883

[48] Demeterco C, Itkin-Ansari P, Tyrberg B, Ford LP, Jarvis RA, et al (2002) c-Myc controls proliferation versus differentiation in human pancreatic endocrine cells. J Clin Endocrinol Metab 87: 3475–3485. 12107268 10.1210/jcem.87.7.8700

[49] Giustiniani J, Daire V, Cantaloube I, Durand G, Poüs C, et al (2009) Tubulin acetylation favors Hsp90 recruitment to microtubules and stimulates the signaling function of the Hsp90 clients Akt/PKB and p53. Cell Signal 21: 529–539. 19136058 10.1016/j.cellsig.2008.12.004

[50] Santander VS, Bisig CG, Purro SA, Casale CH, Arce CA, et al (2006) Tubulin must be acetylated in order to form a complex with membrane Na^+^,K^+^-ATPase and to inhibit its enzyme activity. Mol Cell Biochem 291: 167–174. 16733802 10.1007/s11010-006-9212-9

[51] Aldana-Masangkay GI, Rodriguez-Gonzalez A, Lin T, Ikeda AK, Hsieh YT, et al (2011) Tubacin suppresses proliferation and induces apoptosis of acute lymphoblastic leukemia cells. Leuk Lymphoma 52: 1544–1555. 21699378 10.3109/10428194.2011.570821 PMC4113006

